# Supplementary material for: Differences in sleep spindle wave density between patients with diabetes mellitus and matched controls: implications for sensing and regulation of peripheral blood glucose
Source: medRxiv. 2024 Apr 12:2024.04.11.24305676. Preprint. [Version 1] doi: 10.1101/2024.04.11.24305676 (PMC11030297; doi:10.1101/2024.04.11.24305676)
Supplement: Supplement 1 [file media-1.pdf]

Supplementary materials for

Differences in sleep spindle wave density between patients with diabetes mellitus and matched controls: implications for sensing and regulation of peripheral blood glucose

Supplementary Tables

Table S1. Prevalence of use of prescribed medications and of common complications among diabetes patients and control subjects matched on sex and age.

Diabetic medications	Diabetes (N = 1,214)		Control (N = 1,214)	
	N	%	N	%
DPP-4[1] inhibitors	48	3.9	1	0
GLP-1[2] agonists	306	25.2	15	1.2
Insulin	629	51.8	0	0
Metformin	689	56.7	57	4.6
SGLT2[3] inhibitors	175	14.4	7	0.5
Sulfonylureas	132	10.8	7	0.5
TZDs[4]	15	1.2	2	0.1
Others[5]	10	0.8	3	0.2
Major complications				
Coronary artery disease	257	21.1	81	6.6
Chronic kidney disease	130	10.7	11	0.9
Neuropathy	217	17.8	41	3.3
Stroke	61	5.0	4	0.3
No listed complications	711	58.5	1,086	89.4

[1] dipeptidyl peptidase IV
[2] glucagon-like peptide 1
[3] sodium-glucose transport protein 2
[4] thiazolidinediones
[5] alpha-glucosidase inhibitors, bile acid sequestrants, dopamine-2 agonists, meglitinides

Table S2. Comparison of selected sleep architecture characteristics between patients with diabetes and controls among those who underwent diagnostic PSGs.

Group	N	Min.	1st Qu.	Median	Mean	3rd Qu.	Max.	*P* value[1]
		Total sleep time (min.)						
Diabetes		111.5	306.5	357.5	350.8	405.8	594.0	
Control	1,188	103.0	314.0	365.0	359.9	409.8	654.0	0.009
Diabetes-Control		-390.5	-77.0	-7.5	-9.2	59.5	394.5	
		Sleep efficiency (%)						
Diabetes		20.7	68.4	79.2	76.2	87.4	99.0	
Control	1,210	22.0	70.1	80.2	77.3	87.9	98.5	0.08
Diabetes-Control		-60.9	-12.8	-0.7	-1.1	10.9	57.0	
		Sleep latency (min.)						
Diabetes		0.0	5.3	13.3	23.2	28.6	253.9	
Control	1,210	0.0	6.3	14.4	21.8	27.7	196.7	0.9
Diabetes-Control		-169.8	-14.0	-1.1	1.5	14.6	250.1	
		N2 duration (min. × 2)						
Diabetes		8.5	164.1	211.0	213.2	261.0	463.0	
Control	1,214	30.0	167.6	214.5	211.3	253.5	463.5	0.5
Diabetes-Control		-301.0	-60.9	1.0	2.0	64.4	307.0	
		AHI (events/hour)						
Diabetes		0.0	5.4	11.2	16.2	18.9	90.1	
Control	378	0.0	5.3	9.2	12.8	15.9	71.9	0.009
Diabetes-Control		-57.6	-6.8	1.4	3.4	10.0	88,8	

[1] *P* value from two-sided Wilcoxon signed-rank test comparing diabetes patients to controls subjects; it tests the null hypothesis that the sleep variable specified is the same for diabetes patients and control subjects who underwent diagnostic PSGs.

Table S3. Comparison of six spindle characteristics between patients with diabetes mellitus and control subjects matched on sex and age. Estimates and test based on 1,214 matched patient-control pairs.

Spindle Characteristic	Brain Region	Diabetes		Control		Paired difference		
		Median	IQR	Median	IQR	Median	IQR	*P* value[1]
Duration (sec)	Frontal (F3)	0.75	0.10	0.76	0.09	-0.01	0.14	0.9
	Frontal (F4)	0.74	0.10	0.76	0.09	-0.01	0.14	0.9
	Central (C3)	0.76	0.10	0.78	0.09	-0.02	0.14	0.9
	Central (C4)	0.76	0.09	0.78	0.09	-0.02	0.14	0.9
Absolute Power ($\log_{10} \mu V^2$)	Frontal (F3)	-0.67	0.29	-0.59	0.24	-0.09	0.38	0.8
	Frontal (F4)	-0.64	0.28	-0.57	0.23	-0.07	0.37	0.9
	Central (C3)	-0.63	0.25	-0.56	0.21	-0.08	0.35	0.8
	Central (C4)	-0.62	0.24	-0.55	0.21	-0.07	0.33	0.9
Relative Power	Frontal (F3)	0.30	0.04	0.32	0.04	-0.01	0.05	0.9
	Frontal (F4)	0.30	0.04	0.31	0.04	-0.01	0.05	0.8
	Central (C3)	0.31	0.04	0.32	0.04	-0.01	0.05	0.9
	Central (C4)	0.30	0.04	0.32	0.04	-0.01	0.05	0.9
Frequency (Hz)	Frontal (F3)	12.25	0.55	12.28	0.50	-0.03	0.73	0.8
	Frontal (F4)	12.26	0.53	12.29	0.49	-0.04	0.73	0.8
	Central (C3)	12.62	0.63	12.70	0.63	-0.08	0.81	0.7
	Central (C4)	12.61	0.58	12.69	0.63	-0.06	0.78	0.9
Number of Oscillations	Frontal (F3)	8.64	1.15	8.84	1.05	-0.22	1.56	0.9
	Frontal (F4)	8.60	1.13	8.84	1.10	-0.24	1.63	0.9
	Central (C3)	8.93	1.15	9.17	1.10	-0.25	1.64	0.9
	Central (C4)	8.87	1.12	9.12	1.12	-0.28	1.60	0.8
Symmetry	Frontal (F3)	0.50	0.04	0.50	0.03	1.71e-3	0.06	0.3
	Frontal (F4)	0.50	0.05	0.50	0.03	1.30e-5	0.06	0.4
	Central (C3)	0.50	0.03	0.49	0.03	1.81e-3	0.05	0.2
	Central (C4)	0.49	0.04	0.49	0.03	8.94e-4	0.05	0.1

[1] *P* value from two-sided Wilcoxon signed-rank test applied to paired differences; it tests the null hypothesis that spindle density is the same for diabetes patients and controls subjects.

Table S4. Linear regression analysis of the difference in spindle density (spindles per min) between patients with diabetes and age-and sex-matched control subjects versus average age (yrs) of the matched pair for each brain region separately. Estimates and test based on 1,214 matched patient-control pairs.

EEG channel	Estimated slope	Standard Error	t value	*P* value
F3	-0.0025	0.0027	-0.94	0.3
F4	-0.0018	0.0027	-0.68	0.5
C3	-0.0005	0.0030	-0.18	0.9
C4	-0.0018	0.0028	-0.64	0.5

Supplementary Figures

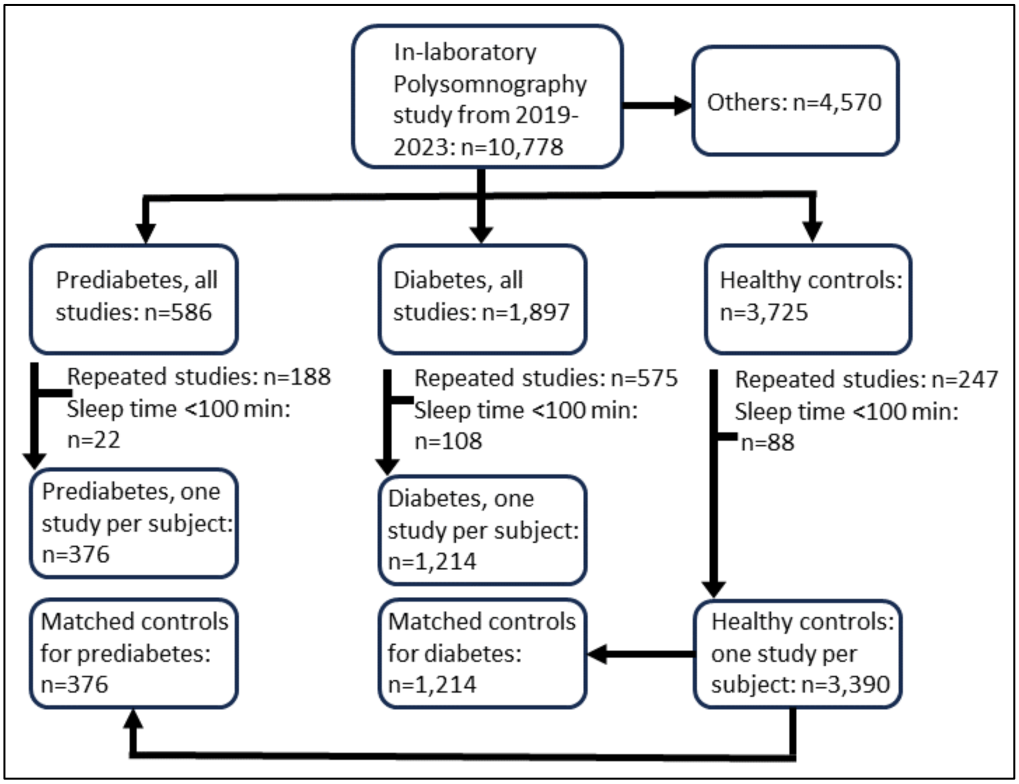

Figure S1. Patient inclusion flowchart.

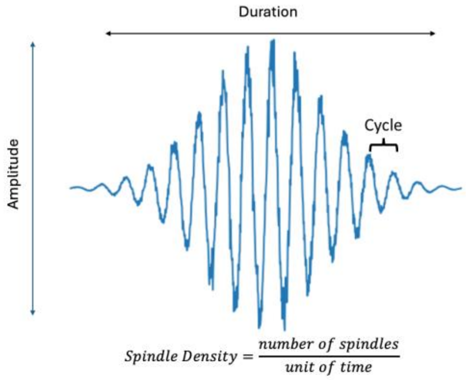

Figure S2. A cartoon illustration of a single spindle.

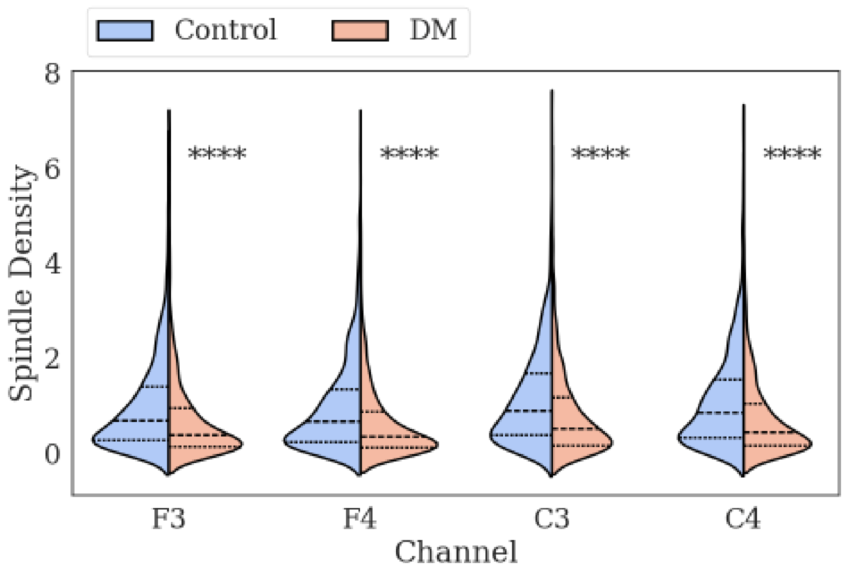

Figure S3. Differences in spindle density between patients with diabetes mellitus and controls. Violin plots (right, diabetes (DM); left, control) of the distribution of the spindle density in the frontal (F3 and F4) and central (C3 and C4) regions of the brain. Dashed lines show medians; dotted lines show the 1st and the 3rd quartiles. **** indicates $P<0.0001$ (two-sided Wilcoxon signed-rank test).

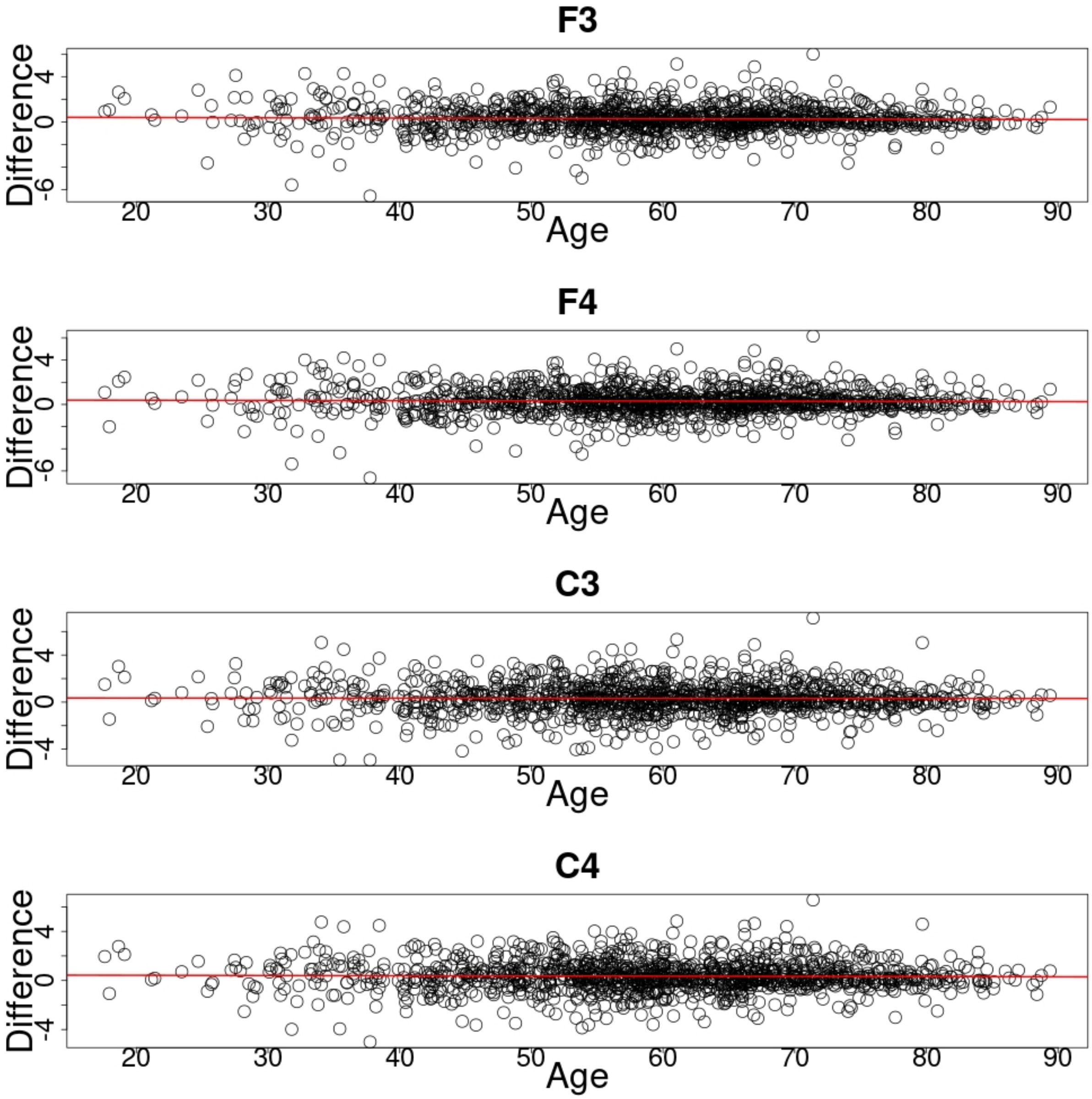

Figure S4. Linear regression analysis of the difference in spindle density (spindles per min) between patients with diabetes and age-and sex-matched control subjects versus average age (yrs) of the matched pair for each brain region separately. Plotted circles represent data for each of the 1,214 matched pairs. The red line is the fitted regression line (**Table S4)**.
